# Dynamic correlation network analysis of financial asset returns with network clustering

**DOI:** 10.1007/s41109-017-0031-6

**Published:** 2017-05-23

**Authors:** Takashi Isogai

**Affiliations:** 1 Bank of Japan, 2-1-1 Nihonbashi-Hongokucho, Chuo-ku, Tokyo, 103-0021 Japan; 2 Tokyo Metropolitan University, 1-4-1 Marunouchi, Chioda-ku, Tokyo, 100-0005 Japan

**Keywords:** Financial asset returns, Correlation network, Dynamic correlation, Network clustering, Dimensionality reduction

## Abstract

In this study, we propose a novel approach to analyze a dynamic correlation network of highly volatile financial asset returns by using a network clustering algorithm to deal with high dimensionality issues. We analyze the dynamic correlation network of selected Japanese stock returns as an empirical study of the correlation dynamics at the market level by applying the proposed method. Two types of network clustering algorithms are employed for the dimensionality reduction. Firstly, several stock groups instead of the existing business sector classification are generated by the hierarchical recursive network clustering of filtered stock returns in order to overcome the high dimensionality problem due to the large number of stocks. The stock returns are then filtered in advance to control for volatility fluctuations that can distort the correlation between stocks. Thus, the correlation network of individual stock returns is transformed into a correlation network of group-based portfolio returns. Secondly, the reduced size of the correlation network is extended to a dynamic one by using a model-based correlation estimation method. A time series of adjacency matrices is created on a daily basis as a dynamic correlation network from the estimation results. Then, the correlation network is summarized into only three representative correlation networks by clustering along the time axis. Some intertemporal comparisons of the dynamic correlation network are conducted by examining the differences between the three sub-period networks. Our dynamic correlation network analysis framework is not limited to stock returns, but can be applied to many other financial and non-financial volatile time series data.

## Introduction

A large number of financial assets including stocks and foreign exchange rates are traded in a financial market. The correlation of individual asset returns (price changes) is one of the key issues to understand the financial market structure. A deeper understanding of the market-wide correlation structure helps us improve financial portfolio management, leading to efficient risk management for investors as well as the authorities responsible for macro financial stability. There is a substantial body of work on correlation networks that analyzed complicated interactions and market structure of financial asset. Mantegna (1999) developed a correlation network of US stock returns by calculating the cross correlation of returns to discover the hierarchical structure of the correlation network. Similar network-based analyses have been conducted including Tumminello et al. (2010), which studied how to obtain hierarchical networks from a correlation matrix of financial asset returns including stock prices; the correlation-based clustering procedure was implemented to explore the hierarchical tree structure of the system. Chi K et al. (2010) also studied a large correlation network that included all US stock returns to examine the interdependence structure of returns. They identified a small number of stocks that has very strong influence on returns of other stocks from their correlation network analysis. Onnela et al. (2003) focused on the dynamics of market correlations. Their study on a time-dependent correlation network of the US stock return data showed that the topological structure of the network is robust with respect to time, while strong market correlation is identified during crisis periods. Preis et al. (2012) also quantified state-dependent correlations in stock markets to know if correlations are not constant but instead vary in time. Their empirical study on major US stocks showed that a higher level of average correlations were observed during market stress periods. More recently, Kenett et al. (2015) applied partial correlation analysis to stock prices by using dependency network to uncover dependency and influence relationship between individual stocks. Their empirical study based on stock prices revealed that one stock can be influenced by different sectors outside of its primary sector classification. They also found that developed markets such as the US, UK, and Japan exhibit higher degree of market stability.

When analyzing the correlation structure at the market level, the number of financial assets can become a serious technical constraint on any empirical analysis. If the number of individual asset is very large as in the case of stock market, the number of pairs of assets may become too large to observe individual relationships between them. Such dimensionality issue together with significantly volatile price movements of financial asset returns should be appropriately controlled when conducting correlation network analysis of financial asset returns. Our previous research (Isogai 2014, 2016) has proposed approaches to overcome such difficulties by applying network theory combined with advanced econometric models. Such a network-based analysis framework is useful to cope with the complex correlation structure between individual asset returns when the interaction of highly volatile asset returns is appropriately considered in network building. Specifically, Isogai (2014) proposed a novel approach to the clustering of a large correlation network by using recursive hierarchical network division. This method is able to find a grouping of highly correlated asset returns that depends only on the adjacency matrix converted from the correlation matrix of filtered returns. Later work also extended the correlation network analysis framework to a dynamic one, in which conditional correlations are estimated to express a dynamically changing network of asset returns (see Isogai (2016)). This method has proven to be useful, especially for the change point detection of the correlation structure.

In this study, we combine the two aforementioned methods to provide summary information on the possible dynamic changes in the correlation structure of a large number of asset returns. Our proposal comprises two types of dimensionality reductions: the first one provides summary information on the group structure of asset returns, while the second provides summary information on the intertemporal differences in the correlation structure. Such a twofold dimensionality reduction is useful for investors and regulatory authorities to find out more about the dynamic changes of a large and complicated financial market. We then apply the proposed method to a large Japanese stock dataset as a typical empirical case study of correlation network analysis.

The remainder of this paper is organized as follows. “Correlation network of financial asset returns” describes the filtering process used to control for the volatility fluctuations of stock returns as well as the network clustering algorithm for filtered returns used to build a reduced-size correlation network at the market level. “Dynamic changes in correlation networks” describes the dynamic conditional correlation (DCC) model used to estimate the time-dependent correlation matrices of stock returns; then, a dynamic correlation network of stock returns is built according to the estimation results of the DCC model. “Comparative analysis of the dynamic correlation network” describes the dimensionality reduction of a time series of adjacency matrices by using low rank tensor decomposition and a subspace clustering algorithm along the time axis. The dynamic correlation network is categorized into three sub-period networks and a comparative analysis is conducted. “Discussion and conclusion” discusses the further enhancement of our methods and possible extension of our dynamic correlation network analysis to other financial and non-financial time series data.

## Correlation network of financial asset returns

A correlation network is a network whose adjacency matrix is built on the basis of pairwise correlations between variables. The interaction between individual stock returns can be regarded as a correlation network whose adjacency matrix is constructed from the correlation matrix of those returns. The nodes of the correlation network are stocks that have edges weighted by the degree of the pairwise correlation of returns. Following the literature, we focus on the contemporaneous co-movement of returns, since this plays a key role in the risk–return relationship of an asset portfolio. The network is, therefore, an undirected and weighted network.

This study aims to establish an efficient way in which to observe possible changes in a large-scale correlation structure of financial asset returns. In financial markets, the correlation between asset prices can change dynamically in response to the trading activities of market participants. The correlation network therefore needs to be extended from a static one to a dynamic one, in which the adjacency matrix changes dynamically depending on time.

However, establishing such a method is beset by difficulties. The first and most important point when building a correlation network is how to estimate the pairwise correlations (edge weights) precisely. The correlation network is based on an observable measure of correlations; many financial time series tend to exhibit volatile features that make carrying out the statistical procedures used to estimate the correlations difficult. If the estimated correlation is distorted, any analysis of the correlation network would be misleading. We thus apply statistical filtering to the return data when calculating the correlation of our returns.

The second problem is how to handle the high dimensionality of financial data. In this study, we use data on Japanese stock returns listed on the Tokyo Stock Exchange (TSE). More than 1,700 stocks are listed on the First Section of the TSE; the total number of all listed stocks is more than 3,000 in Japan. The first dimensionality issue arising from the number of assets is that it is hard to handle such a large correlation matrix.

Another dimensionality problem is related to the observation frequency of price data, which determines the temporal resolution of dynamic change detection. More frequent price data enable a more precise analysis of dynamic changes; however, this increases the dimensionality alongside the time axis. To deal with these two high dimensionality issues, dimensionality reduction by clustering the correlation network in terms of size and time is implemented, respectively. We begin by describing the filtering method used to calculate the correlations of volatile financial asset returns in the following section.

### Filtering volatile asset returns

Many financial asset returns have fat-tailed return distributions in that large-scale volatility shocks are observed frequently as discussed in many previous research works including Mandelbrot (1963) and Cont (2007). A market-wide shock such as a financial crisis as well as smaller shocks that affect part of the market can distort the calculation of asset returns from sample data, as discussed in Isogai (2014, 2016). Such synchronized volatility shocks between multiple asset returns can cause an overestimation of the correlation. It is therefore crucial to control for the volatility fluctuations of asset returns when calculating the correlation matrix, which is converted into an adjacency matrix later.

Isogai (2014) filtered asset returns to remove volatility fluctuations, using an econometric volatility model, namely the generalized autoregressive conditional heteroskedasticity (GARCH) model proposed by Bollerslev (1986). Asset returns comprise two parts: the conditional mean and shock as follows:

1 $$ \begin{aligned} &\boldsymbol{r}_{t}=\boldsymbol{\mu}_{t}+\boldsymbol{\varepsilon}_{t}=\boldsymbol{\mu}_{t}+\boldsymbol{H}_{t}^{1/2}\boldsymbol{z}_{t},\\ &\mathrm{E}\left(\boldsymbol{z_{t}}\right)=\boldsymbol{0},\ \text{Var}\left(\boldsymbol{z_{t}}\right)=\boldsymbol{I}_{N}\\ \end{aligned}  $$

where ***r***_*t*_ is a vector of the asset returns, ***μ***_*t*_ is a vector of the conditional means, ***ε***_*t*_ is a vector of the unpredictable residuals, ***H***_*t*_ is an *N*×*N* (*N*: the number of returns) symmetric positive-definite matrix, which is a conditional variance–covariance matrix of ***r***_*t*_, and ***z***_*t*_ is a vector of the i.i.d. standardized residuals, the mean and variance of which are ***0*** and ***I***_*N*_ (an identity matrix of order *N*), respectively.

More specifically, the mean part is modeled by using the autoregressive moving average (ARMA) model independently as

2 $$ \boldsymbol{\mu}_{t}=\boldsymbol{\mu}+\sum\limits_{i=1}^{P} \boldsymbol{A}_{i} \boldsymbol{r}_{t-i} +\sum\limits_{j=1}^{Q} \boldsymbol{B}_{j} \boldsymbol{\varepsilon}_{t-j}  $$

where ***A***_*i*_ and ***B***_*j*_ are diagonal matrices. The volatility part is modeled as

3 $$ \boldsymbol{h}_{t}=\boldsymbol{\omega}+\sum\limits_{i=1}^{q}\boldsymbol{S}_{i}\boldsymbol{\varepsilon}_{t-i} \odot \boldsymbol{\varepsilon}_{t-i}+\sum\limits_{j=1}^{p}\boldsymbol{T}_{j}\boldsymbol{h}_{t-j}  $$

where ⊙ denotes the Hadamard operator (the entry-wise product), ***h***_*t*_ is the diagonalized matrix of ***H***_*t*_, and both ***S***_*i*_ and ***T***_*j*_ are diagonal matrices. Volatility is modeled without interaction between the assets to simplify the model.

What is important in (1) is that the volatility fluctuation can be separated from returns ***r***_*t*_ as the elements of ***h***_*t*_ such as $\left (\sqrt {h_{11{\cdot }t}},\ \ldots,\ \sqrt {h_{NN{\cdot }t}}\right)$. Thus, we can safely estimate the linear correlation of returns ***r***_*t*_ as correlation matrix ***R*** by calculating the sample pairwise correlation of filtered residuals ***z***_*t*_, since ***h***_*t*_ as well as ***μ***_*t*_ have no effect on the Pearson linear correlation coefficient of ***r***_*t*_ defined as $\rho _{r_{i},r_{j}}$ =$\frac {\text {Cov}\left (r_{i},r_{j}\right)}{\sqrt {\text {Var}\left (r_{i}\right)\cdot \text {Var}\left (r_{j}\right)}}$. Note that we use a static correlation matrix that is assumed to be constant during the observation period at this stage.

When fitting the model to our dataset, we assume that the distribution of individual residual *z*_*i*_ is a normal, Student *t*, or skew *t* distribution, allowing for some fat-tailedness even after the volatility filtering. The model parameters are estimated as maximum likelihood estimators (MLEs) for each asset independently, since the model does not include any interaction between the assets as shown in (2) and (3). This estimation process works efficiently, especially with a large number of assets. We have now established a way in which to overcome the distortion problem of the linear correlation caused by volatility fluctuations when estimating the correlation of volatile asset returns. For more technical details on the estimation procedures, see Isogai (2014).

Next, we build a static correlation network from the estimated correlation matrix ***R*** by using a simple unsigned nondecreasing adjacency conversion formula with thresholding based on ***R*** as follows:

4 $$ \boldsymbol{A}_{ii}=0, \quad \boldsymbol{A}_{ij\left(i\neq j\right)}= \left\{\begin{array}{ll} \left|cor\left(x_{i},\, x_{j}\right)\right| &\quad if\ \,\, cor\left(x_{i},\, x_{j}\right)>cor_{thres(i, j)}\\ 0 &\quad if\ \,\, cor\left(x_{i},\, x_{j}\right)\leqq {cor}_{thres(i, j)}\\ \end{array}\right.  $$

where ***A***_*ij*_ is the (*i,j*)_*th*_ entry of weighted adjacency matrix ***A***; *cor*(*x*_*i*_, *x*_*j*_) is the (*i,j*)_*th*_ entry of correlation matrix ***R***; and *cor*_*thres*(*i, j*)_ is the cutoff point of the edge weight. All the diagonal elements of ***A***_*ij*_ are 0, since no self-edge is considered in the correlation network. An undirected weighted network only with edges positively weighted is built by (4). The cutoff point *cor*_*thres*(*i, j*)_ is set at the higher level of the two 20th percentiles of the empirical edge weight distribution of stocks *i* and *j*. In other words, *cor*(*x*_*i*_, *x*_*j*_) is set at 0 when correlation *cor*(*x*_*i*_, *x*_*j*_) is lower than the lower 20th percentile of *cor*(*x*_*i*_, ·) or that of *cor*(·,*x*_*j*_). The threshold level is sufficiently high to exclude correlation values that are not statistically meaningful from our dataset. It is confirmed that the clustering result has not been much affected by thresholding at lower levels.

### Dataset for the empirical analysis

As mentioned in “Introduction”, we focus on Japanese stock return data as an empirical case study. The dataset used covers the stocks listed on the First Section of the TSE: 1,324 stocks in 33 business sectors. Note that stocks with low liquidity are excluded from the dataset. The observation period runs from January 2008 to May 2016 (2,058 trading days). The study period includes major two financial crises: the Lehman collapse (2008) and the Great East Japan Earthquake (2011). Stock returns are calculated by using daily closing data as log returns.

We fit the GARCH model, expressed by (1), (2), and (3), to those individual stock return data to calculate correlation matrix ***R*** from the filtered returns. Then, the static correlation network of individual stock returns is created by the adjacency conversion, as shown in (4).

### Grouping by recursive network division

The network built in the last section is too large to carry out correlation analysis; more than 1,300 nodes are densely connected with many other nodes. Hence, we need to conduct the first-round dimensionality reduction of the correlation network as mentioned earlier. The whole stock market is regarded as a market portfolio in which every stock is included. This portfolio can be separated into several sub-portfolios; then, the correlation structure of the whole market is approximated by the correlations among sub-portfolios. What is important here is how to organize a grouping of stock returns. The most frequently used approach for grouping stock returns is to adopt a predefined industrial sector classification. The business industry classification is adopted in the TSE; however, the sector classification is not necessarily consistent with the observed correlation structure of stock returns. Furthermore, such a sector classification tends to be significantly unbalanced in size, as discussed by Isogai (2014).

**Fig. 1:**
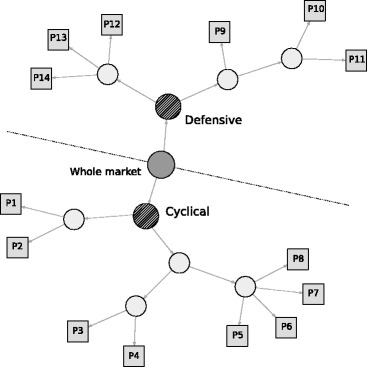
Network clustering by recursive hierarchical network division. Note: The whole market includes all 1,324 stocks as a single group portfolio. The network is divided into several sub-networks (marked by *circles*) so as to maximize the modularity of the network. The two major categories: Cyclical and Defensive groups are created at the first division of the whole market. This modularity based network division is applied to the sub-networks recursively until the final 14 groups (marked by *squares*) are created. Further details of the recursive division algorithm is described in Isogai (2014)

To address these shortcomings, Isogai (2014) proposed a data-driven method for the classification of stock returns based on a recursive network division algorithm. Under this method, hierarchical sub-networks are created by dividing the correlation network recursively. In particular, the network division method used in this study employs the modularity maximization to generate the optimal classification of stock returns at every stage of the recursive network division, as shown in Fig. 1. First, we apply a graph spectral clustering algorithm with the modularity optimization to adjacency matrix ***A*** defined in (5) of the whole correlation network of individual stock returns. The modularity *Q*, proposed by Girvan and Newman (2002) and Newman (2006), of ***A*** is defined as

5 $$ \begin{aligned} Q&=\frac{1}{2W}\sum\limits_{i=1}^{n} \sum\limits_{j=1}^{n}{B_{ij}} \delta \left({C_{i},C_{j}} \right) \\ w_{i}&=\sum\limits_{j=1}^{n} {w_{ij}}, 2W=\sum\limits_{i=1}^{n} {w_{i}}, B_{ij}={\left({A_{ij} -\frac{w_{i} w_{j} }{2W}} \right)} \\ \end{aligned}  $$

where *w*_*i*_,*w*_*j*_ are the sum of the weights of stock *i*, *j*; *δ*() takes 1 if both stocks are in the same class (*C*_*i*_=*C*_*j*_), otherwise 0; and *Q* takes the value between -1 and 1 with positive values indicating the possible presence of some group structure. We employ the simplest definition from among the many variants of modularity definitions. Once the first level of the division is completed by the modularity optimization, the same algorithm is applied to the generated groups of stock returns to make further divisions recursively. As for the stopping rule of recursive division, standard deviations of edge weights in individual groups are monitored to determine the number of groups; the group division process is controlled so as to avoid any significant heterogeneity in terms of group size that can cause heavy concentration of stocks in specific groups. A more detailed explanation of the recursive network division algorithm is described in Isogai (2014).

Finally, the hierarchical group structure is identified as having 14 unit groups (marked as squares with labels P1, P2, …, and P14), as shown in Fig. 1. The circles in Fig. 1 indicate the whole market and intermediate groups created in the layers in-between. Two major categories, termed Cyclical and Defensive, are created at the first division of the entire network. These two categories are used as the broadest categorization of the Japanese stock market for the following comparative analysis. The groups labeled P1, P2, …, and P14 are the unit group portfolios used to approximate the correlation structure of the whole stock market. Each group includes stocks of different sizes, which are categorized as either Cyclical or Defensive.

Table 1 shows more detailed summary information about the network clustering result. The Cyclical and Defensive groups have a 55 and a 45 percent share of all stocks, respectively. The Cyclical group has eight sub-groups labeled from P1 to P8, which are the same as those shown in Fig. 1. The Defensive group has six sub-groups labeled from P9 to P14. Three other features of those groups are listed there: the TOPIX (market capitalization-weighted stock price index) beta and exchange rate (Dollar/Yen) beta as price responsiveness to these variables, company size index, and overseas sales ratio. Despite their similar sizes and the magnitudes of the company size index, there are some distinctive differences between the Cyclical and Defensive groups. The betas are clearly higher in the Cyclical group than the Defensive group, while significant differences exist within the groups of the two categories. The combination of higher betas with a higher overseas sales ratio in the Cyclical group and lower betas with a lower overseas sales ratio in the Defensive group seem to be convincing, since the Cyclical group is more export-oriented, while the Defensive group is more dependent on domestic demand. This information is helpful to understand the features of the groups created by network clustering.

**Table 1:** Clustering result with group features

			Beta			
Group id	Number of stocks	(share, %)	TOPIX	Exchange rate	Company size index	Overseas sales ratio
Cyclical	728	(55.0)	1.01	1.08	62.9	50.3
P1	141	(10.6)	0.93	0.93	35.3	47.1
P2	181	(13.7)	0.87	0.92	35.4	36.6
P3	62	(4.7)	1.01	1.16	63.5	34.6
P4	132	(10.0)	0.90	0.95	43.0	42.9
P5	54	(4.1)	1.08	1.19	72.9	61.6
P6	62	(4.7)	1.12	1.15	81.3	66.5
P7	52	(3.9)	1.05	1.10	79.1	50.8
P8	44	(3.3)	1.11	1.21	92.6	61.9
Defensive	596	(45.0)	0.74	0.75	59.1	29.1
P9	164	(12.4)	0.90	0.99	64.6	38.5
P10	75	(5.7)	0.61	0.60	27.3	30.0
P11	92	(6.9)	0.70	0.72	40.8	27.1
P12	118	(8.9)	0.83	0.89	81.5	31.5
P13	62	(4.7)	0.73	0.71	75.6	25.5
P14	85	(6.4)	0.69	0.62	64.8	21.9

Note: Group ids from P1 to P14 are identified in Fig. 1. The major categories of Cyclical and Defensive groups are defined as the result of the first binary division of the whole universe of stocks as described in Fig. 1. The data of the two major categories are group mean values. Beta is calculated by a robust MM-estimator for individual stocks with one factor (TOPIX and US dollar/Japanese yen exchange rate, respectively) linear model as the elasticity of stock price change to the factor change; then, averaged as the group mean value. Company size index is the percentile value of empirical distribution of market capitalization of individual stocks. Overseas sales ratio is simply averaged over stocks which have the data for each group

Table 2 shows the business sector breakdown of the individual groups. The sectors that have the highest three shares are listed for each group. In the Cyclical group, Transportation equipment and Electric appliances, which are typical export-oriented sectors, are included. In the Defensive group, Foods, Electric power and gas, and Services are typical domestic demand-dependent sectors. The list of sectors shown there seems to be consistent with the comparison of group features in Table 1. Some sectors including Services appear in both the Cyclical and the Defensive categories. In the case of the Services sector, software and IT-related companies are included in Cyclical, while healthcare-related companies are included in Defensive, for example. Such a division within a single sector seems to be realistic, however. Indeed, a similar type of within-sector division is also partly observed in the Cyclical and Defensive categories. Hence, we assume that our network clustering created a reasonable set of sub-portfolios that reflect the business features of stock groups in Japan. One caveat of the grouping of stocks is that it has not been clarified why and how such groups are formed in the stock market. We need to examine a wider range of data for further analysis in this regard, which is beyond the scope of this paper.

**Table 2:** Business sector breakdown of identified groups (top three sectors)

Sector breakdown (share %)							
Group id		(a)		(b)		(c)	(a+b+c)
Cyclical							
P1	Electric appliances	17	Services	12	Machinery	10	39
P2	Construction	18	Machinery	13	Wholesale trade	10	41
P3	Securities	21	Other financial business	13	Real estate	11	45
P4	Electric appliances	20	Chemicals	18	Wholesale trade	16	54
P5	Transportation equipment	39	Electric appliances	20	Machinery	11	70
P6	Electric appliances	47	Machinery	23	Chemicals	10	80
P7	Chemicals	19	Iron and steel	17	Nonferrous metals	13	49
P8	Electric appliances	30	Transportation equipment	18	Chemicals	9	57
Defensive							
P9	Banks	26	Construction	11	Chemicals	9	46
P10	Retail trade	19	Information and communication	17	Wholesale trade	17	53
P11	Retail trade	17	Wholesale trade	16	Foods	8	41
P12	Information and communication	17	Foods	15	Retail trade	14	46
P13	Electric power and gas	26	Pharmaceutical	16	Foods	15	57
P14	Retail trade	53	Services	19	Information and communication	8	80

Note: Only the three largest sectors are listed for each group. The standard business sector classification comprises 33 industrial sectors, which is widely used as the standard sector classification in Japan

Next, the sub-portfolios based on the classification of those Cyclical and Defensive groups are created and averaged price index returns are calculated for each group. More specifically, the stock price of a sub-portfolio is first indexed as 100 at the beginning of the observation period; then, the mean value of the sub-portfolio is calculated with an equal weight placed on each stock. The portfolio return is defined as the log return of the mean value of the portfolio as in the case of individual stock returns. The market portfolio including more than 1,300 stocks is now summarized into only 14 sub-portfolios by using the group definition provided by network clustering.

**Fig. 2:**
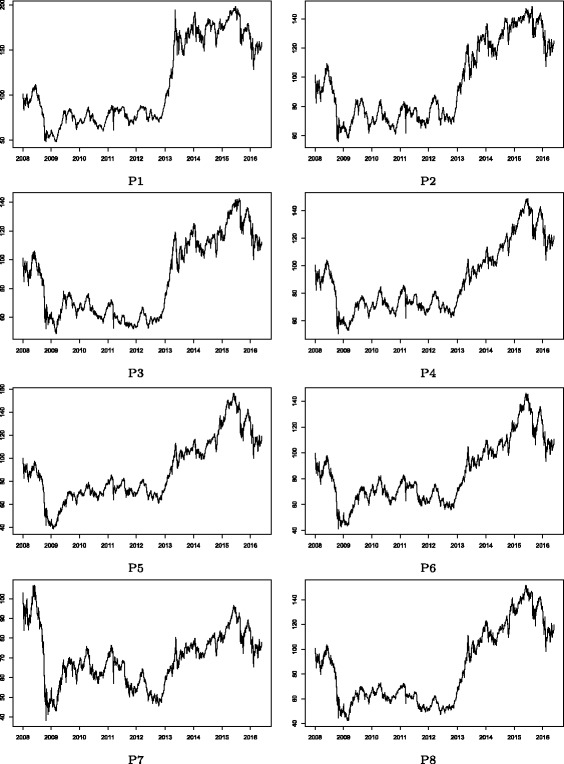
Stock price indices: Cyclical group sub-portfolios. Note: The sub-groups of P1, P2, …, and P8 correspond to the same group ids shown in Fig. 1. The stock price index of a sub-portfolio is first indexed as 100 at the beginning of the observation period (January 2008); then, the log returns of the mean value of the portfolio is calculated. The price index figures of sub-groups appear to be similar to each other because of the market-wide common price co-movements in the stock market. Some local temporal differences between sub-groups still exist, reflecting local group-specific common factors

**Fig. 3:**
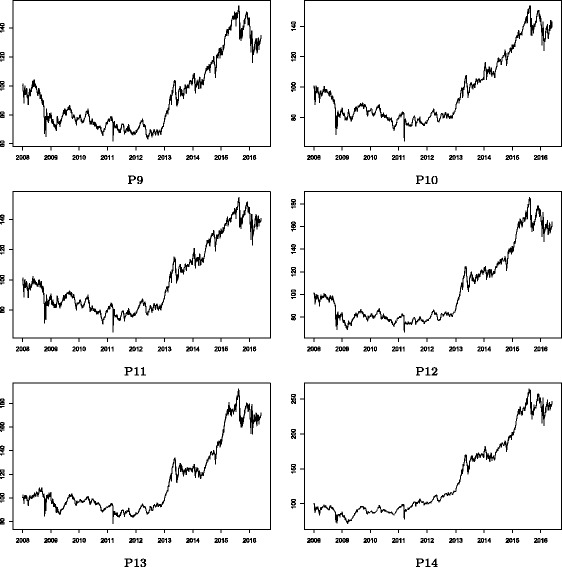
Stock price indices: Defensive group sub-portfolios. Note: The sub-groups of P9, P10, …, and P14 correspond to the same group ids shown in Fig. 1. The stock price index of a sub-portfolio is first indexed as 100 at the beginning of the observation period (January 2008); then, the log returns of the mean value of the portfolio is calculated. The price index figures of sub-groups appear to be similar to each other because of the market-wide common price co-movements in the stock market. Some local temporal differences between sub-groups still exist, reflecting local group-specific common factors

**Fig. 4:**
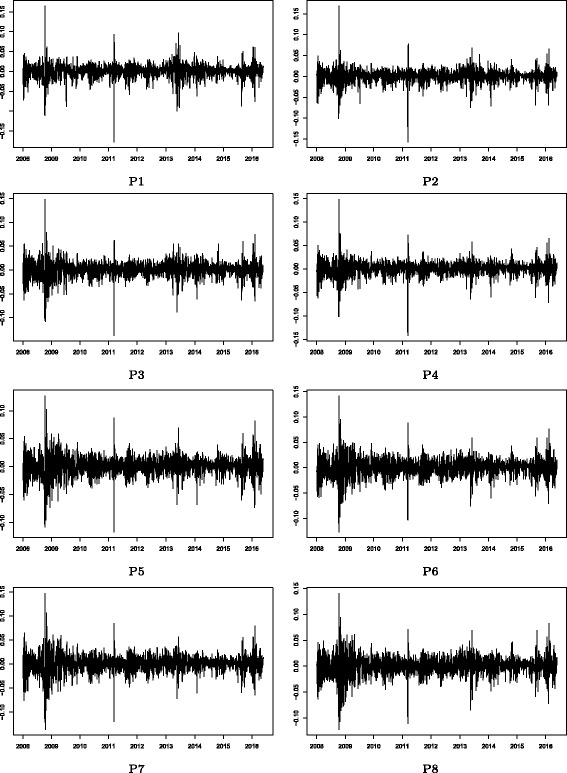
Stock returns: Cyclical group sub-portfolios. Note: The stock return of a sub-portfolio is calculated as the daily log return of the stock price index of the group-based sub-portfolio shown in Fig. 2

**Fig. 5:**
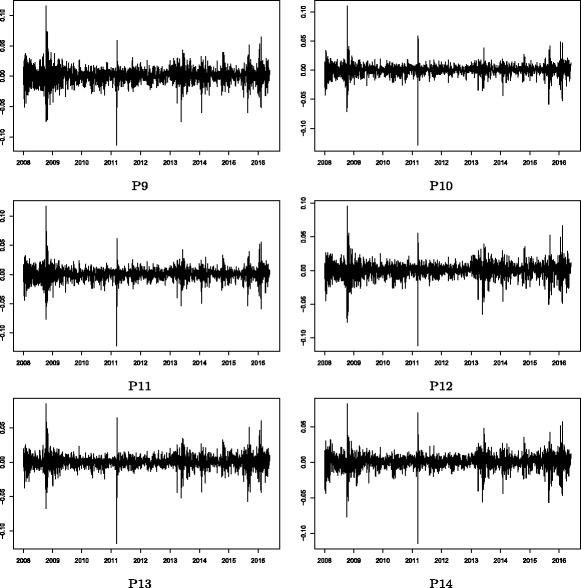
Stock returns: Defensive group sub-portfolios. Note: The stock return of a sub-portfolio is calculated as the daily log return of the stock price index of the group-based sub-portfolio shown in Fig. 3

Figures 2 and 3 show the stock price indices of the Cyclical and Defensive group-based sub-portfolios, respectively. Note that these price indices appear to follow roughly the same trend in both figures. This is mainly because of the market-wide common price co-movements in the stock market. There are, however, some clear differences observed in local temporal changes in price indices between these groups, which suggest that group-specific common factors drive such temporal divergences from the market-wide movement. Figures 4 and 5 show the log returns calculated from the stock price indices shown in Figs. 2 and 3, respectively. Significant volatility changes are detected in every sub-portfolio as mentioned in “Filtering volatile asset returns”. These changes necessitate using the filtering process by employing the GARCH model in advance of the estimation of the return correlation. Further, there are two sharp volatility increases (price plunges) in 2008 (the Lehman collapse) and in 2011 (the Great East Japan Earthquake). Those two events caused financial market turbulence that affected the entire Japanese stock market. However, we are interested in whether the market-wide correlation structure changed during such crisis periods, which is difficult to detect by studying just a single correlation network during the whole observation period. A more advanced method of dynamic correlation network analysis is therefore required.

Thus, the first dimensionality reduction in terms of the number of stocks is completed. Stocks connected with thicker edges (higher correlations) are grouped by network clustering with the modularity optimization; then, the log returns of those group-based sub-portfolios are calculated. Now, we can proceed onto the next stage. In this stage, we extend the static correlation model to a dynamic one followed by the second-round dimensionality reduction of the correlation structure between the group portfolios along the time axis in order to carry out the intertemporal comparative analysis.

## Dynamic changes in correlation networks

In the previous section, we assumed that there exists a static correlation network in which the edges between nodes (stocks) do not change during the observation period. Such an assumption is introduced partly because of technical reasons regarding the estimation of the correlation matrix of stock returns. It is rather an empirical issue whether the correlation structure is stable or dynamically changing over time. The linkages between the nodes may change because of changes in the correlation of stock returns, although it is not technically easy to detect such dynamic changes from observed price data. Normally, a sample pairwise correlation of returns is calculated based on the observed filtered or unfiltered return data; therefore, only one sample correlation, the static one, is available for one data period. However, it would be meaningful to know how the correlation network changes over time from the viewpoint of investment decision making as well as portfolio risk control if we could establish a method of estimating dynamic correlations.

In this context, Isogai (2016) proposed a novel approach to build a dynamic correlation network of returns. We adopt a model-based correlation instead of using a filtered sample correlation to calculate the correlations and adjacency matrices for network clustering. This model-based correlation matrix can be estimated for each trading day during the observation period and a dynamic correlation network built accordingly. A series of adjacency matrices are calculated by adjacency conversion from the estimated correlation matrices. The dimensionality issue due to the large number of adjacency matrices needs to be addressed to allow an intertemporal comparison of the network. We overcome this issue by using the second-round dimensionality reduction of the adjacency matrices.

### Model-based dynamic correlation

The most frequently used approach to measure the dynamic correlations of asset returns is to calculate the correlations of returns over moving windows. A series of correlation matrices can be built by using a rolling observation time window during the study period. This method, however, has some drawbacks as discussed in Isogai (2016). Hence, a statistical model-based correlation can be used to describe the dynamic correlation network. The DCC model originally proposed by Engle (2002) was developed in the context of using multivariate volatility models in financial econometrics. The static unconditional correlation matrix ***R*** of return ***r***_*t*_ is calculated as the correlation matrix of filtered return ***z***_*t*_ defined in (1) when building the static correlation network. When applying the DCC model, a time-dependent correlation matrix ***R***_*t*_ of return ***r***_*t*_ is estimated instead of ***R***. This means that the correlation of returns can change dynamically during the observation period; therefore, dynamic changes in the structure of a correlation network are represented by a dynamic conditional adjacency matrix ***A***_*t*_. There are multiple adjacency matrices, as many as the number of trading days converted from a series of ***R***_*t*_ during the period.

The DCC model is described as an extension of the multivariate GARCH model described by (1), (2), and (3). Specifically, an *N*×*N* positive-definite dynamic correlation ***R***_*t*_ is introduced to model the dependency structure of ***r***_*t*_. The time dependency of ***R***_*t*_ is described by using a proxy variable ***Q***_*t*_, which is introduced to ensure the positive-definiteness of ***R***_*t*_ as

6 $$ \begin{aligned} \boldsymbol{Q}_{t} &=\boldsymbol{\bar{Q}}+\sum\limits_{i=1}^{m}a_{i}\left(\boldsymbol{z}_{t-i}\boldsymbol{z}_{t-i}^{'}-\boldsymbol{\bar{Q}}\right)+\sum\limits_{j=1}^{n}b_{j}\left(\boldsymbol{Q}_{t-i}-\boldsymbol{\bar{Q}}\right)\\ \end{aligned}  $$

where *a*_*i*_ and *b*_*j*_ are non-negative scalars and $\boldsymbol {\bar {Q}}_{t}$ is the unconditional mean of ***Q***_*t*_. The DCC model with time lags in the conditional correlation is denoted as DCC (*m, n*). The parameter *a*_*i*_ shows the sensitivity of ***Q***_*t*_ to previous shocks, while the parameter *b*_*j*_ represents the persistence of the correlation in previous periods. The correlation matrix ***R***_*t*_ is calculated by rescaling ***Q***_*t*_ as

7 $$ \boldsymbol{R}_{t}=\text{diag}\left(\boldsymbol{Q}_{t}\right)^{-\frac{1}{2}}\boldsymbol{Q}_{t}\text{diag}\left(\boldsymbol{Q}_{t}\right)^{-\frac{1}{2}}  $$

8 $$ a_{i} \ge 0,\quad b_{j} \ge 0, \quad \sum\limits_{i=1}^{m}a_{i}+\sum\limits_{j=1}^{n}b_{j}<1.  $$

For more details on the DCC–GARCH model, see Engle and Sheppard (2001) and Engle (2002).

### Model fitting and adjacency conversion

The parameters of the DCC model are estimated by using MLEs with the Japanese stock return data. We employ a two-stage fitting of the DCC model: the first stage of the GARCH model fitting followed by the second stage of the DCC parameter estimation. The GARCH model is fitted to the sub-portfolio returns just as it is fitted to individual stock returns in “Correlation network of financial asset returns”. Once the first stage of the model estimation is completed, the filtered residuals ***z***_*t*_ of 14 sub-portfolios are calculated by using the estimated parameters of the GARCH model. In the second stage, the DCC model parameters used to calculate the dynamic correlation ***R***_*t*_ of the sub-portfolio returns are estimated.

When estimating the DCC parameters by using MLEs, the joint density function of ***r***_*t*_ should be explicitly defined in advance. The distribution of ***z***_*t*_ is assumed to be one of the normal, Student *t*, or skew *t* distributions again. The joint density function *f*(***r***_*t*_) is then defined by using a copula density function that determines the dependency between the sub-portfolio returns. The copula function plays a key role in addressing the dependency between the heterogeneous distributions of ***z***_*t*_ as mentioned above. In general, the joint density function *f*(*x*) of a vector of variable ***X***=(*X*_1_,…,*X*_*N*_) can be described using a copula function as follows:

9 $$ \begin{aligned} f\left(x_{1},\,\ldots,\, x_{N}\right) &=c\left(F_{1}\left(x_{1}\right),\,\ldots,\, F_{N}\left(x_{N}\right)\right)\prod_{i=1}^{N}f_{i}\left(x_{i}\right) \end{aligned}  $$

where *f*_*i*_(*x*_*i*_) is the marginal distribution of *x*_*i*_,*c*(·) is the density function of the copula, and *F*(·) is the joint distribution function of ***X***. We choose the Student *t*-copula that can handle tail dependency, which takes two parameters: conditional correlation ***R***_*t*_ and the constant shape parameter. For technical details on the copula and Student *t*-copula, see Sklar (1959) and Demarta and McNeil (2005). Thus, the joint density function of ***r***_*t*_ is defined as a combination of the copula density and density of the i.i.d. residual ***z***_*t*_:

10 $$ \begin{aligned} &f\left(\boldsymbol{r}_{t}|\boldsymbol{\mu}_{t},\ \boldsymbol{h}_{t},\ \boldsymbol{R}_{t},\ \eta\right)\\ &=c^{S_{t}}\left(u_{1{\cdot}t},\ \ldots,\,\ u_{N{\cdot}t}|\boldsymbol{R}_{t},\ \eta\right) \prod_{i=1}^{N}\frac{1}{\sqrt{h_{i{\cdot}t}}}f_{i{\cdot}t}\left(z_{i{\cdot}t}|\theta_{i}\right) \end{aligned}  $$

where *u*_*i*·*t*_=*F*_*i*_(*r*_*i*·*t*_|*μ*_*i*·*t*_,*h*_*i*·*t*_,*θ*_*i*_); *θ*_*i*_ is a parameter set including the ARMA–GARCH parameters in (2) and (3) and the distributional parameters of *z*_*i*_; $\phantom {\dot {i}\!}c^{S_{t}}(\cdot)$ is the Student *t*-copula density function; and *η* is the shape parameter of the Student *t*-copula. The conditional correlation ***R***_*t*_ is defined as one parameter of the copula function, the time-dependent structure of which is described in (6) and (7) in the DCC model setting. The estimate of ***R***_*t*_ therefore collapses to the non-negative scalars (***a***, ***b***) defined in (6).

We need to determine the distribution of ***z***_*t*_ as well as the DCC order (*m, n*): the model selection is made by comparing the goodness-of-fit measure, namely the Akaike information criterion (AIC), from the multiple combinations of the model settings. The log-likelihood function *LL*(***θ***|***r***_*t*_) built by using (10) comprises two parts: the copula part with the DCC parameters (***a***, ***b***) and marginal distribution part *f*_*i*·*t*_(*z*_*i*·*t*_|*θ*_*i*_). The two parts of the log-likelihood function can be maximized independently: first, the individual distributional parameter set *θ*_*i*_ is estimated, followed by the DCC parameters (***a***, ***b***). More technical details about the model fitting procedures are described in Isogai (2016); Patton (2006), and Joe (2005).

Table 3 shows the parameter estimation results for the selected DCC models. The DCC order (*m, n*) is (1, 2); *a*_1_, the sensitivity of the correlation to previous shocks, takes a small value. The larger value of *b*_1_+*b*_2_ means that the dynamic correlation matrix ***R***_*t*_ is more dependent on its past values than previous shocks, since the parameter *b*_*j*_ represents the degree of persistence of the correlation. The model parameter restriction shown in (8) is confirmed to be satisfied. The shape parameter of the Student-*t* copula is not so low, meaning that the degree of tail dependency seems to be limited after volatility filtering. The other details of the estimation results including the ARMA–GARCH model of individual returns are omitted because of space limitations.

**Table 3:** DCC parameter estimation results

	m, n	a1	b1	b2	[b1+b2]	*η*
Estimate	1, 2	0.0259	0.5549	0.3855	[0.9404]	13.0
(*P*-value)		(0.0000)	(0.0000)	(0.0000)		(0.0000)

Note: The DCC order (*m, n*) and parameters *a*_1_, *b*_1_, and *b*_2_ are defined in (6). *η* is the shape parameter of the Student *t*-copula in (10). The R (http://cran.r-project.org/) package “rmgarch” Ghalanos (2014) is used for the parameter estimation

The same DCC model is fitted to the individual stock returns in each sub-portfolio to examine the extent to which the correlation of the individual stock returns are significantly different between sub-portfolios. Table 4 shows the DCC parameter estimation results for all sub-portfolios. The DCC order (*m, n*) is (1, 2) for all sub-portfolios in Cyclical, while it is (1, 2) or (1, 1) in Defensive. The combination of lower *a*_1_ and higher *b*_1_+*b*2 values appears to be common to every portfolio, while the relative share of the two types of parameters varies over the sub-portfolios. Again, the shape parameter of the Student-*t* copula is higher in every sub-portfolio. This result means that the dynamic correlation properties are similar, although small differences exist between the sub-portfolios. Further, we can extend our dynamic correlation network analysis to those sub-portfolios when required.

**Table 4:** DCC parameter estimation results by sub-portfolio

		m, n	a1	b1	b2	[b1+b2]	*η*
Cyclical	P1	1, 2	0.0080	0.5537	0.3523	[0.9060]	30.5
	P2	1, 2	0.0074	0.5567	0.3792	[0.9358]	19.7
	P3	1, 2	0.0093	0.3275	0.3897	[0.7172]	29.9
	P4	1, 2	0.0079	0.5476	0.3219	[0.8695]	31.6
	P5	1, 2	0.0064	0.5787	0.3240	[0.9027]	27.2
	P6	1, 2	0.0086	0.5820	0.3219	[0.9038]	22.3
	P7	1, 2	0.0079	0.5432	0.3713	[0.9145]	25.6
	P8	1, 2	0.0069	0.5542	0.3925	[0.9467]	20.9
Defensive	P9	1, 2	0.0078	0.4651	0.3815	[0.8467]	27.9
	P10	1, 2	0.0103	0.2498	0.3980	[0.6478]	30.9
	P11	1, 2	0.0070	0.4963	0.3890	[0.8853]	30.9
	P12	1, 1	0.0048	0.9400	-	[0.9400]	22.9
	P13	1, 2	0.0094	0.2627	0.5084	[0.7711]	27.1
	P14	1, 1	0.0042	0.8945	-	[0.8945]	38.5

Note: The DCC order (*m, n*) and parameters *a*_1_,*b*_1_, and *b*_2_ are defined in (6). *η* is the shape parameter of the Student *t*-copula in (10). The R (http://cran.r-project.org/) package “rmgarch” Ghalanos (2014) is used for the parameter estimation

Next, we build a dynamic correlation network to study the possible topological changes in the network. The DCC parameter estimates are all available; the dynamic correlation matrix from the estimated correlation matrix ***R***_*t*_ can be easily calculated from (6). Then, the estimated model-based conditional correlation matrix ***R***_*t*_ is converted into the conditional adjacency matrix of the dynamic correlation network. Here, we use the same unsigned nondecreasing adjacency conversion as the one in (4) used when building the static correlation network for clustering stock returns in “Correlation network of financial asset returns”. The adjacency conversion is extended to a time-dependent conditional one as follows:

11 $$ \boldsymbol{A}_{ii,t}=0, \quad \boldsymbol{A}_{ij\left(i\neq j\right),t}= \left\{\begin{array}{ll} \left|cor\left(x_{i},\, x_{j}\right)_{t}\right|\ &\quad if\ \,\, cor\left(x_{i},\, x_{j}\right)_{t} > {cor}_{thres(i, j)_{t}}\\ 0 & \quad if\ \,\, cor\left(x_{i},\, x_{j}\right)_{t}\leqq {cor}_{thres(i, j)_{t}}\\ \end{array}\right.  $$

where ***A***_*ij,t*_ is the (*i, j*)_*th*_ entry of the conditional weighted adjacency matrix ***A***_*t*_ and *cor*(*x*_*i*_, *x*_*j*_)_*t*_ is the (*i, j*)_*th*_ entry of the dynamic correlation matrix ***R***_*t*_. The diagonal element of ***A***_*ij,t*_ is 0. The threshold value $\phantom {\dot {i}\!}{cor}_{{thres}({i, j})_{t}}$ is determined in the same way as in (4) at every point in time. Thus, the dynamic correlation network is created with the adjacency matrices ***A***_*t*_ available for each trading day. One technical issue is that thresholding of the adjacency matrix entries can affect the result of our intertemporal analysis described below. The thresholding method is time-dependent; therefore, threshold values of the same matrix entry can change dynamically. It may cause discontinuous changes, especially when the threshold level is higher. These aspects of our dynamic thresholding method makes it difficult to forecast its effect on intertemporal analysis. We confirmed that analytical results are stable with thresholding in a few different settings; however, this point is still an important caveat of this study.

## Comparative analysis of the dynamic correlation network

In the previous section, a dynamic correlation network of stock returns was successfully built and conditional adjacency matrices ***A***_*t*_ were identified for every trading day. We discuss how to implement the second-round dimensionality reduction of ***A***_*t*_ in this section. The dynamic correlation network represents the time-varying pairwise correlations between the index returns of 14 sub-portfolios. The nodes of the network are those sub-portfolios generated by the network clustering of the overall static correlation network that includes every stock as a node. The index return of each sub-portfolio can be regarded as a factor that jointly determines the whole market movement. Hence, the relationships (edges) between factors (nodes) describe the time-varying relationships between individual stock returns that belong to different sub-portfolios in a reduced dimension: from 1,324 stocks to 14 return indices. The dynamic correlation network carries summary information of the correlation structure of stock returns in the form of conditional adjacency matrix ***A***_*t*_.

In addition to the above-mentioned first-round dimensionality reduction of the number of nodes, we need to reduce the number of adjacency matrices, since it is difficult to compare the adjacency matrices of 2,058 trading days directly. In this context, the second-round dimensionality reduction is introduced by clustering the conditional adjacency matrices. Specifically, we use the subspace clustering of matrices by using the low rank tensor approximation method. Once the adjacency matrices are sorted into a small number of groups, we build reduced-size sub-period adjacency matrices to summarize information on the inter-period changes of the dynamic network.

### Clustering the dynamic adjacency matrices

When clustering adjacency matrices, the conversion of an adjacency matrix into a feature vector needs to be implemented first to apply any clustering algorithm. Some approximation of the original features is often adopted for data compression as well as noise reduction. For this purpose, tensor decomposition is useful with the use of a clustering algorithm such as *k*-means. A tensor is represented as a multidimensional array relative to a choice of the basis of the particular space on which it is defined (for details, see Kroonenberg (2008)). Intuitively, a tensor is a higher-order generalization of vectors and matrices. The conditional adjacency matrices can thus be regarded as a tensor of order three; an adjacency matrix can be similarly regarded as an order two tensor. Principal component analysis (PCA) is often combined with a clustering algorithm when the target data are arranged in a vector form. Eigenvalue decomposition or singular value decomposition (SVD) is then used to decompose a stacked data matrix into several factors. The idea of such decomposition can be extended to tensor-based factor decomposition.

Suppose we have an order three tensor ***X*** that is equivalent to a time series of conditional adjacency matrix ***A***_*t*_. In the tensor-based decomposition, the tensor is represented as the product of some components in the same way as in PCA or SVD. Several tensor decomposition methods have been proposed including canonical polyadic decomposition (CP) as described in Carroll and Chang (1970) and Bro (1997), Tucker decomposition proposed by Tucker (1966), and higher-order SVD (HOSVD) by Lathauwer et al. (2000). We use the Tucker decomposition method to group the adjacency matrices, which offers a flexible choice of lower rank decomposition. For more detailed information about low rank tensor approximation, see Kolda and Bader (2009) and Grasedyck et al. (2013).

In the Tucker decomposition, $\boldsymbol {X}\in \boldsymbol {\mathcal {R}}^{n_{1}\times n_{2}\times n_{3}}$ is decomposed into factor matrices with orthogonal columns $\boldsymbol {U}_{k}\in \boldsymbol {\mathcal {R}}^{n_{k}\times r_{k}}$ (*k*=1, 2, and 3) and core tensor $\boldsymbol {\mathcal {G}}\in \boldsymbol {\mathcal {R}}^{r_{1}\times r_{2}\times r_{3}}$ as

12 $$ \boldsymbol{X}=\boldsymbol{\mathcal{G}}\boldsymbol{\times}_{1}\boldsymbol{U}_{1}\boldsymbol{\times}_{2}\boldsymbol{U}_{2}\boldsymbol{\times}_{3}\boldsymbol{U}_{3}  $$

where *n*_1_ and *n*_2_ correspond to the size of conditional adjacency matrix ***A***_*t*_; *n*_3_ is the length of observation period (the number of trading days); ***×***_1_, ***×***_2_, and ***×***_3_ are tensor products in the corresponding mode; *r*_1_,*r*_2_, and *r*_3_ are lower ranks for the approximation in each direction. In our dataset, *n*_1_(= *n*_2_) is 14; *n*_3_ is 2,058.

The rank order selection of (*r*_1_,*r*_2_,*r*_3_) is flexible in the Tucker decomposition. We set *r*_1_=*r*_2_=3 for the further dimension reduction of the conditional adjacency matrix, while we set *r*_3_=*n*_3_=2,058 to preserve information about changes along the time axis as much as possible for time series clustering. Thus, the time series of adjacency matrices are decomposed into three orthogonal unit factors and one core tensor.

Next, we perform the subspace clustering of adjacency matrices, using the result of the Tucker decomposition. We focus on the subspace spanned by ***U***_3_, which is the orthogonal basis for the time horizon, since we are mainly interested in the differences between trading days. The projection of ***X*** onto the subspace is defined as

13 $$ \boldsymbol{Y}=\boldsymbol{\mathcal{G}}\boldsymbol{\times}_{3}\boldsymbol{U}_{3}  $$

***Y*** is then transformed into a vector used as the feature vector for clustering by *k*-means. As for the choice of *k*, we calculate the gap statistics (Tibshirani et al. 2001) for a different number of clusters (*k*) to find the best value. The gap statistics analysis indicates that the best *k* is around 4; however, we select *k*=3 to simplify the intertemporal comparison.

The time series of conditional adjacency matrix ***A***_*t*_ are categorized into three groups in terms of trading date by *k*-means clustering with low rank tensor decomposition. The clustering result means that the dynamically changing correlation network is classified into three types. In other words, the whole observation period can be divided into three sub-periods, in which the network has a different correlation structure. The time series of the adjacency matrices are projected onto only the three representative adjacency matrices in a way that minor differences between them are discarded to highlight the major differences. The second-round dimensionality reduction is thus achieved, allowing us to make an intertemporal comparison of the correlation structure.

### Classification of dynamic correlation networks

**Fig. 6:**
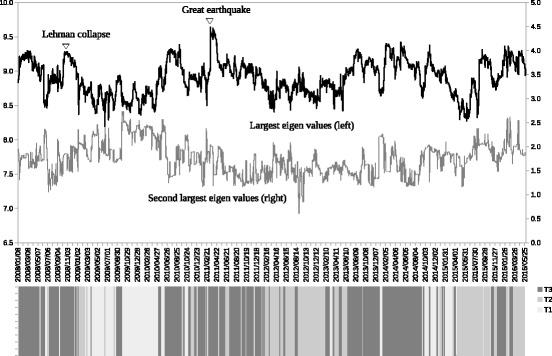
Sub-period classification result of the dynamic correlation network. Note: The largest and the second largest eigenvalues of the adjacency matrix of the dynamic correlation network of sub-portfolio returns are plotted at each trading day as the *two solid lines* on the *top*. The clustering result of the conditional adjacency matrices during the whole observation period are shown as the *shaded bar* graph on the *bottom*. T1, T2, and T3 represents the three sub-periods as the three division of the whole period as shown in Tables 5 and 6

Figure 6 shows the result of the sub-period classification with the largest and second largest eigenvalues of the adjacency matrices. Every trading date from the beginning of January 2008 to the end of May 2016 is labeled as one of T1, T2, and T3 and shaded in different colors at the bottom of Fig. 6. The three classes of sub-periods are clearly identified as local blocks on the time axis. A change from one of the three sub-periods to another can be regarded as a phase shift of the dynamic correlation network.

**Table 5:** Largest eigenvalue index by sub-period

	Mean		Max		Min	
	Index	(Index-100)	Index	(Index-100)	Index	(Index-100)
T1	98.56	(-1.44)	96.73	(-3.27)	101.16	(+1.16)
T2	98.83	(-1.17)	97.14	(-2.86)	100.00	(0.00)
T3	102.14	(+2.14)	100.00	(0.00)	104.18	(+4.18)
Whole period	100		100		100	
[value]	[8.90]		[9.64]		[8.19]	

Note: Every trading date during the whole observation period (January 2008 - May 2016) was separated into three sub-periods, namely T1, T2, and T3 by the subspace clustering of conditional adjacency matrices of the dynamic correlation network using *k*-means algorithm. Mean, Max, and Min represent the mean, maximum, and minimum values of the largest eigenvalues of adjacency matrices that are estimated on each trading date during the sub-period, respectively. The values are indexed at the whole period=100

**Table 6:** Topological features of the correlation network by sub-period

	Density		Centralization		Heterogeneity	
T1	0.625		0.149		0.273	
T2	0.614		0.158		0.313	
T3	0.638		0.147		0.304	
Whole period	0.627		0.151		0.282	
	Index	(Index-100)	Index	(Index-100)	Index	(Index-100)
T1	99.66	(-0.34)	99.09	(-0.91)	96.65	(-3.35)
T2	97.96	(-2.04)	104.76	(+4.76)	110.78	(+10.78)
T3	101.75	(+1.75)	97.47	(-2.53)	107.52	(+7.52)
Whole period	100		100		100	

Note: T1, T2, and T3 represent the sub-periods identified by the subspace clustering of conditional adjacency matrices as shown in Table 5. The relative indices of those three topological measures are indexed as 100 at the whole period levels, respectively

Intuitively, the largest eigenvalue of an adjacency matrix represents the strength of the correlation in the network. Higher levels of the largest eigenvalues are observed during the crisis periods including the Lehman collapse (2008) and the Great East Japan Earthquake (2011), which means a stronger linkage between nodes. The levels of the largest eigenvalues seem to be somewhat related to the sub-period type.

Table 5 shows the relative changes to the largest eigenvalue, which is indexed at the whole period=100. The mean values of the sub-periods show that T3 has a higher level of the largest eigenvalue compared with T1 and T2. The maximum value during the whole period exists in T3 as shown by the 100 value in the column Max, while the minimum value exists in the column Min. The comparison of the largest eigenvalue confirms that T3 is a stress period, while T1 and T2 are normal periods.

**Fig. 7:**
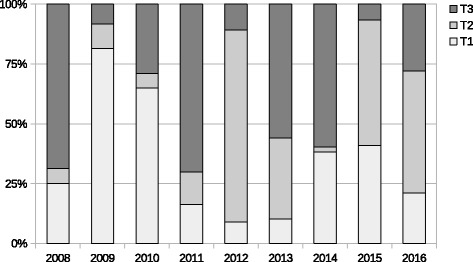
Sub-period breakdown of the dynamic correlation network. Note: The shaded areas of an individual bar represent the shares of three sub-period categories, T1, T2, and T3, in each year. The share is calculated as the ratio of number of trading days of each category to the total trading days in the year

Figure 7 shows the sub-period breakdown of the trading dates during the observation period in terms of T1, T2, and T3. The share of each sub-period is greatly different depending on the year. Looking at the share of T3, a larger share of T3 is observed in 2008, 2011, 2013, and 2014. As mentioned earlier, large financial shocks happened in 2008 (the Lehman collapse) and 2011 (the Great East Japan Earthquake) when the sub-period T3 has a higher share. A fundamental change in Japanese monetary policy (i.e., quantitative monetary easing) happened in 2013 and 2104, which caused market-wide stock price rallies at that time. T3 also has a higher level of shares in those periods. Those explicit differences in terms of the trading date share of T3 also confirm that T3 seems to be a stressed sub-period when the linkages of the correlation network intensified, as suggested by the analysis in Fig. 6 and Table 5. There are some differences between T1 and T2, although both periods appear to be normal as mentioned earlier. Figure 7 shows that T1 has higher shares in 2009 and 2010, while T2 has higher shares in 2012, 2015, and the first half of 2016. Those explicit differences in terms of the trading date share suggest that it is meaningful to observe T1 and T2 separately.

Table 6 shows the topological features of the correlation network in the three sub-periods. The selected three topological measures of conditional adjacency matrix ***A***_*t*_ are density (*D*), centralization (*C*), and heterogeneity (*H*). The three measures are defined as

14 $$ D(\boldsymbol{A}_{t}) = \frac{{\sum\nolimits}_{i}{\sum\nolimits}_{j>i}\boldsymbol{A}_{t,ij}}{n\left(n-1\right)/2}\approx\frac{mean\left(\boldsymbol{k}_{t}\right)}{n}, \quad k_{t,i}=\sum\limits_{j\neq i}\boldsymbol{A}_{t,ij}  $$

15 $$ \begin{aligned} C(\boldsymbol{A}_{t})&=\frac{n}{n-2}\left(\frac{max\left(\boldsymbol{k}_{t}\right)}{n-1}-D(\boldsymbol{A}_{t})\right)\approx\frac{max\left(\boldsymbol{k}_{t}\right)}{n}-D(\boldsymbol{A}_{t})\\ \end{aligned}  $$

16 $$ H(\boldsymbol{A}_{t})=\frac{\sqrt{var\left(\boldsymbol{k}_{t}\right)}}{mean\left(\boldsymbol{k}_{t}\right)}=\sqrt{\frac{n\sum_{i}k_{t,i}^{2}}{\left(\sum_{i}k_{t,i}\right)^{2}}-1}  $$

where *n* is the number of nodes; ***k***_*t*_ is a vector of the node degree (connectivity) defined as the sum of the row or column of an adjacency matrix; and *max*(·),*mean*(·), and *var*(·) are the maximum, mean, and variance function, respectively. For more details on these topological measures, see Horvath (2011). Density *D*, defined as the mean of the off-diagonal elements of ***A***_*t*_, measures the overall connection (correlation) among nodes: a density close to 1 indicates that all nodes are strongly correlated with each other. Centralization *C* is 1 when one node has fully connected edges with all other nodes that are not connected with each other; 0 when each node has the same connectivity. Heterogeneity *H*, defined as the coefficient of variation of the connectivity distribution, measures the variation in connectivity across nodes. These three topological measures are calculated for conditional adjacent matrix ***A***_*t*_ for every trading day during the observation period and then summarized as mean values and indices in Table 6.

The comparison of the network topological measure indices between sub-periods in Table 6 provides further information to understand the characteristics of the three sub-periods. Stressed period T3 has a higher level of density and heterogeneity, but lower centralization. As for the two normal periods, T1 and T2 have a different combination of topological features. In T1, all three measures are at a lower level with no significant change from the whole period average. T2 has a lower level of density, but higher centralization and heterogeneity than the average. However, it is difficult to know any more from such a network topology comparison. T1 and T2 as well as those two and T3 have different topological features with regard to the dynamic correlation network, reconfirming that the three-period classification is sufficiently meaningful for further comparative analysis, although we need more data to delve into the detail.

### Intertemporal changes of the correlation networks

The division into three sub-periods described in the last section enables us to reduce the large dimension of conditional adjacency matrix ***A***_*t*_ from 2,058 (trading days) to only three. This dimensionality reduction simplifies the comparison of the dynamic correlation network on the time axis, allowing us to summarize the differences between so many networks in comparison with the three networks that represent the corresponding sub-period. Specifically, the adjacency matrices for each sub-period are simply averaged for each entry of the matrix to create an adjacency matrix that represents the corresponding sub-period. Finally, the dynamic correlation structure of the entire Japanese stock market is summarized by using only the three 14-by-14 adjacency matrices. We can therefore make pairwise comparisons of these three networks.

**Fig. 8:**
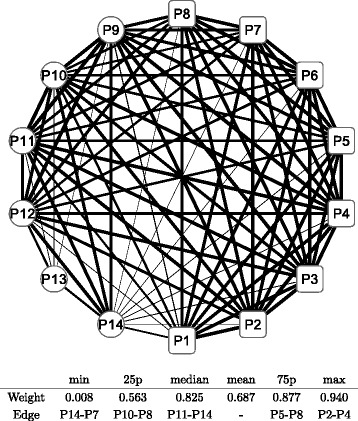
Correlation network of sub-portfolios: whole period. Note: The network is created based on the network adjacency matrix of the whole observation period. The *square* nodes (labeled as P1, P2, …, and P8) represent Cyclical sub-portfolios; the *circle* nodes (labeled as P9, P10, …, and P14) represent Defensive sub-portfolios. The edge between nodes represents the degree of correlation of log returns between the two nodes. The edge width represents the weight of the corresponding entry of network adjacency matrix. The *bottom* table shows the quantiles of edge weights distribution with corresponding edges

**Fig. 9:**
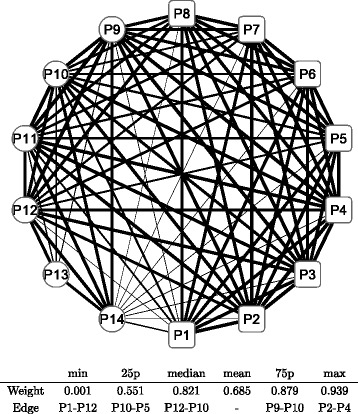
Correlation network of sub-portfolios: sub-period T1. Note: The network is created based on the network adjacency matrix of the sub-period category T1. For further details, see the note of Fig. 8

**Fig. 10:**
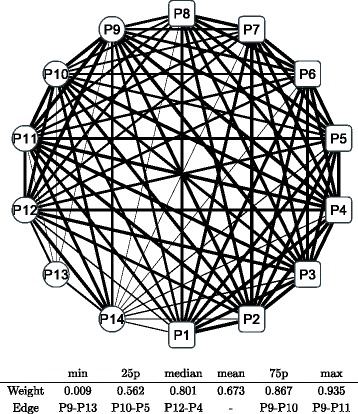
Correlation network of sub-portfolios: sub-period T2. Note: The network is created based on the network adjacency matrix of the sub-period category T2. For further details, see the note of Fig. 8

**Fig. 11:**
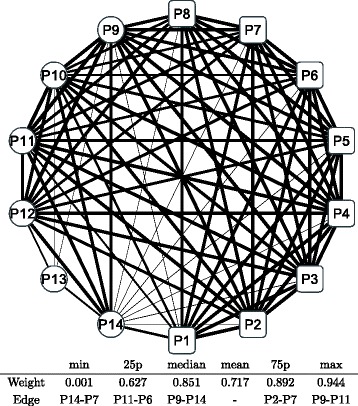
Correlation network of sub-portfolios: sub-period T3. Note: The network is created based on the network adjacency matrix of the sub-period category T3. For further details, see the note of Fig. 8

Figure 8 shows a graphical representation of the correlation network during the whole period; the conditional adjacency matrices are averaged to create the corresponding adjacency matrix. The nodes indicate sub-portfolios as labeled from P1 to P14; a node in a square means the Cyclical group portfolio, while a node in a circle means the Defensive one. The width of the edge represents the edge weight of an adjacency matrix: a thicker edge means a higher level of correlation between the two connected nodes. The network is densely connected with thicker edges, which means that most factors of Japanese stock returns are highly correlated. The connection is denser in the Cyclical group (square nodes), while the connection appears to be less dense in the Defensive group (circle nodes), especially at nodes P13 and P14. The main sector of the two nodes are Electric power and gas, Pharmaceutical, and Foods for P13 compared with Retail trade, Services, and Information and communication for P14. Similarly, we depict graphical representations for the correlation networks in the three sub-periods: T1 in Fig. 9, T2 in Fig. 10, and T3 in Fig. 11. Although slight differences between the three networks exist, the graphical representations of the three sub-period networks appear to be largely the same as the whole period network. In other words, the correlation network of Japanese stock returns remains largely stable, whereas intertemporal changes in the correlation network may also exist.

**Fig. 12:**
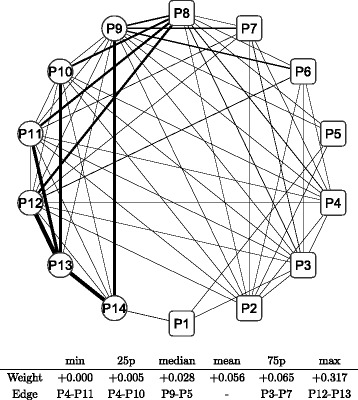
Changes in network between sub-periods: T1–T2. Note: The network shows the difference between the sub-period T1 and T2. The edge weight (the edge width) is calculated by subtracting elements of the adjacency matrix of T2 from those of T1 described as T1 −T2. Only the edges that have positive weights (T1 > T2) are shown. The bottom table shows the quantiles of edge weights distribution with corresponding edges

**Fig. 13:**
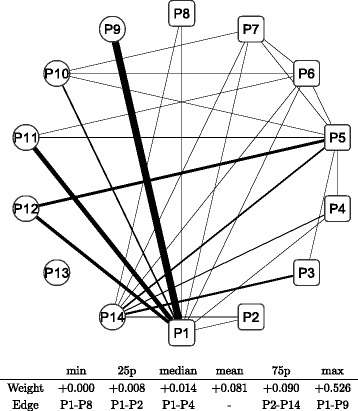
Changes in network between sub-periods: T2–T1. Note: The network shows the difference between the sub-period T2 and T1. The edge weight (the edge width) is calculated by subtracting elements of the adjacency matrix of T1 from those of T2 described as T2 −T1. Only the edges that have positive weights (T2 > T1) are shown. The bottom table shows the quantiles of edge weights distribution with corresponding edges

In order to detect intertemporal changes between the three sub-period networks, another type of network is built based on the adjacency matrix, which is defined as the difference between two adjacency matrices. We have three pairs of sub-periods: T1 and T2, T2 and T3, and T3 and T1. Hence, three adjacency matrices are calculated for each pair of sub-periods. Figures 12 and 13 depict the difference in sub-period networks between T1 and T2 in the same graphical format as in Figs. 8, 9, 10 and 11. The difference between the two is just the direction of the subtraction. Figure 12 shows the sub-period network in which the adjacency matrix of T2 is subtracted from that of T1, denoted as T1–T2. Only edges with a positive weight (correlation) are shown to highlight the increased and decreased edges separately. The network of T1–T2 has thicker edges within Defensive (circle) groups, which means that the correlation between Defensive sub-portfolios increases when the correlation network moves from T2 to T1. Conversely, Fig. 13 shows the sub-period network of T2–T1. Thicker edges appear between the Cyclical groups (specifically, P1, P3, and P5) and Defensive groups (P9, P11, and P12). It means that the correlation over the two major categories increases between those specific nodes when the network moves from T1 to T2.

**Fig. 14:**
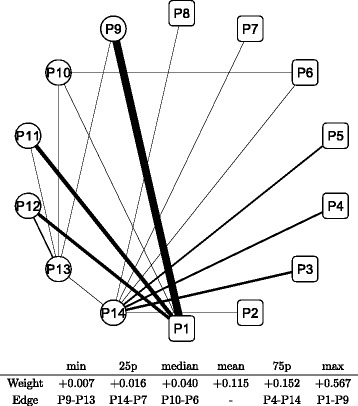
Changes in network between sub-periods: T2–T3. Note: The network shows the difference between the sub-period T2 and T3. The edge weight (the edge width) is calculated by subtracting elements of the adjacency matrix of T3 from those of T2 described as T2 −T3. Only the edges that have positive weights (T2 > T3) are shown. The bottom table shows the quantiles of edge weights distribution with corresponding edges

**Fig. 15:**
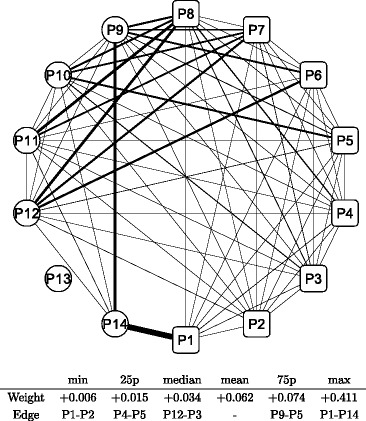
Changes in network between sub-periods: T3–T2. Note: The network shows the difference between the sub-period T3 and T2. The edge weight (the edge width) is calculated by subtracting elements of the adjacency matrix of T2 from those of T3 described as T3 −T2. Only the edges that have positive weights (T3 > T2) are shown. The bottom table shows the quantiles of edge weights distribution with corresponding edges

Figures 14 (T2–T3) and 15 (T3–T2) depict the difference in sub-period networks between T2 and T3. In Fig. 14, thicker edges are observed between the Cyclical groups (specifically, P1 followed by P3, P4, and P5) and Defensive groups (P9, P11, P12, and P14). There are few significant increases observed among the Cyclical group nodes and Defensive group nodes, respectively. In Fig. 15, the thickest edge is observed between P1 and P14 over the two major categories. Some thicker edges are also observed between the Cyclical groups (P6, P7, and P8) and Defensive groups (P9, P10, P11, and P12) as well as within Defensive groups (P9–P14).

**Fig. 16:**
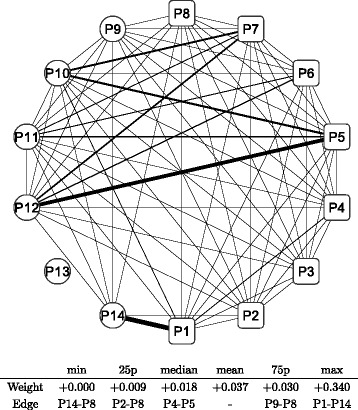
Changes in network between sub-periods: T3–T1. Note: The network shows the difference between the sub-period T3 and T1. The edge weight (the edge width) is calculated by subtracting elements of the adjacency matrix of T1 from those of T3 described as T3 −T1. Only the edges that have positive weights (T3 > T1) are shown. The bottom table shows the quantiles of edge weights distribution with corresponding edges

**Fig. 17:**
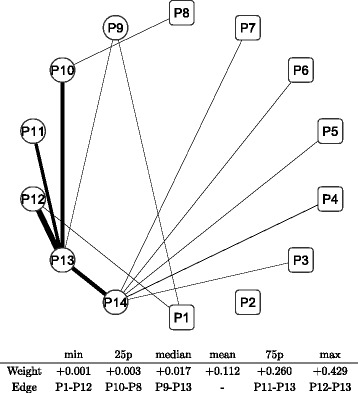
Changes in network between sub-periods: T1–T3. Note: The network shows the difference between the sub-period T1 and T3. The edge weight (the edge width) is calculated by subtracting elements of the adjacency matrix of T3 from those of T1 described as T1 −T3. Only the edges that have positive weights (T1 > T3) are shown. The bottom table shows the quantiles of edge weights distribution with corresponding edges

Figures 16 (T3–T1) and 17 (T1–T3) depict the difference in sub-period networks between T3 and T1. In Fig. 16, two thicker edges (P5–P12 and P1–P14) are clearly observed between the Cyclical groups and Defensive groups. In Fig. 17, thicker edges are observed only within the Defensive group nodes, where P13 is located at the center. No significant increase is observed between the Cyclical group nodes and Defensive group nodes as well as within the Cyclical group nodes.

The changes between T3 and T1 or T2 represents a transition from (to) a normal period to (from) a stressed period. A higher level of correlation is observed during stressed periods as mentioned earlier. The changes shown by Figs. 14, 15, 16 and 17 indicate that there are positive and negative contributions of pairwise correlations between individual groups to the overall intensified correlation during a stressed period. It is also apparent that the pattern of changes to (from) a stressed period is greatly different, reflecting the significant difference of correlation structure between the two normal periods shown by Figs. 12 and 13.

Thus, the correlation network of Japanese stock returns is summarized into a correlation network of 14 sub-portfolio returns by using network clustering; then, the dynamic changes in the network are also summarized into only three static networks to facilitate an intertemporal comparison. Here, we summarize findings from comparative analysis of the three sub-period correlation networks. The correlation network appears to be largely stable over time, while an elevated level of overall correlation are observed during stressed periods (T3) compared with normal periods (T1 and T2). The pairwise comparisons between three sub-periods correlation networks reveal that changes in correlation are observed more clearly within the Defensive groups and between the Cyclical and Defensive groups, whereas changes in correlation within the Cyclical groups are rather limited. The result suggests that there is some fundamental difference in terms of changing pattern of network structure between the two major categories.

## Discussion and conclusion

The dynamically changing correlation network of individual financial returns has been recognized an important topic. There are many research works studied in this regard; however, many of them adopted static correlation measures calculated over a sample period. Intertemporal analysis was often based on a time series of such static correlations calculated by rolling sample periods, which may lead to a biased estimate of correlation as discussed in Isogai (2016). The large number of financial assets causes another technical difficulty when dealing with the correlation network, while the existing sector classification is not reliable for grouping of stocks. Thus, an efficient and reliable method for dimensionality reduction is required for an extended research of correlation network. Dimensionality reduction of a time series of conditional correlation network was another difficult issue for intertemporal comparative analysis. The proposed analytical framework of dynamic correlation network with non-sector based grouping of stock returns can handle these issues in a systematically organized way.

In this study, we proposed a new approach to enable an intertemporal analysis of a large-scale correlation network of financial asset returns in a compact way by combining our previous research works. The main contribution of this study is the provision of two types of dimensionality reduction methods: (i) the reduction of a large correlation network into a smaller factor correlation network and (ii) the reduction of a time series of a correlation network into a countable number of representative correlation networks. Such twofold dimensionality reduction works well to extract important information from complicated correlation networks.

The proposed method, however, is still at an early experimental stage and several issues must be addressed to enhance its efficiency and stability. Firstly, our method heavily depends on econometric time series models including GARCH and DCC models, which are greatly complicated; many simplified assumptions have been introduced in model building. We also need to select a model from many alternatives before undergoing the time-consuming parameter estimation process. Specifically, the DCC model has many technically difficult issues regarding the parameter estimation. As for network-building process, our simple adjacency conversion formula can be improved to enhance the signal–noise separation performance. In addition, many alternative options exist for the selection of the clustering algorithm. Noteworthily, the empirical results of the dynamic correlation network of Japanese stock returns depend on those simplified assumptions and these results may be affected by changing any of the model assumptions. Secondly, the empirical results reveal the need for supplementary analysis to clarify what causes intertemporal changes in the correlation network. The dynamic network analysis only provide an initial clue to identify when and how the network changes; we need more information to enhance our understanding of the meaning of such changes.

With regard to the possible practical application of our proposed method, it can be easily applied to portfolio optimization and risk control in financial investment decisions. In standard financial model settings, the static correlation of returns is normally assumed as one of the key inputs. Even if it is difficult to change the modeling framework fundamentally, our proposed method can thus provide important information about the dynamics of the correlation structure, which contributes to having some appropriate adjustments in the model application. For example, the degree of correlation between the Cyclical and Defensive group portfolios can change significantly between sub-periods as mentioned in the previous section. Such information is greatly helpful for making decisions on investment allocation as well as risk control since ordinary models do not consider such facts.

Lastly, our method can be extended to cover other financial and non-financial time series data with large-scale volatility fluctuations. Not only financial time series other than stock prices tend to have significant volatility fluctuations; therefore, there is a higher chance of applying our model. We may find a different dynamic correlation structure if our method is applied to those time series. Our method is also applicable to non-financial volatile time series, although the careful examination of such extended use of the method is required in advance.

In future research, we will aim to apply our method to non-Japanese stock returns to understand whether a similar result is obtained. The stock market is globally linked closely; we are greatly interested in the dynamic correlation network analysis of stock returns between multiple countries. Further, the dynamic correlation network analysis between different classes of financial assets is another interesting topic for future analysis.
